# Targeting carbohydrate metabolism in colorectal cancer - synergy between DNA-damaging agents, cannabinoids, and intermittent serum starvation

**DOI:** 10.18632/oncoscience.611

**Published:** 2024-11-12

**Authors:** Viktoriia Cherkasova, Olga Kovalchuk, Igor Kovalchuk

**Affiliations:** ^1^Department of Biological Sciences, University of Lethbridge, Lethbridge, Alberta T1K 3M4, Canada

**Keywords:** colorectal cancer, cisplatin, cannabidiol, intermittent serum starvation, chemotherapy

## Abstract

Chemotherapy is a therapy of choice for many cancers. However, it is often inefficient for long-term patient survival and is usually accompanied by multiple adverse effects. The adverse effects are mainly associated with toxicity to normal cells, frequently resulting in immune system depression, nausea, loss of appetite and metabolic changes. In this respect, the combination of chemotherapy with cannabinoids, especially non-psychoactive, such as cannabidiol, cannabinol and other minor cannabinoids, as well as terpenes, may become very useful. This is especially pertinent because the mechanisms of anticancer effects of cannabinoids on cancer cells are often different from conventional chemotherapeutics. In addition, cannabinoids help alleviate chemotherapy-induced adverse effects, regulate sleep and appetite, and are shown to have analgesic properties. Another component for achieving potential anti-cancer synergism is regulating nutrient availability and metabolism by calorie restriction and intermittent fasting in cancer cells. As tumours require a lot of energy to grow and because glucose is constantly available, malignant cells often opt to use glucose as a primary source of ATP production through substrate-level phosphorylation (fermentation) rather than through oxidative phosphorylation. Thus, periodic depletion of cancer cells of primary fuel, glucose, could result in a strong synergy in killing cancer cells by chemo- and possibly radiotherapy when combined with cannabinoids.

This commentary will discuss what is known about such combinatorial treatments, including potential mechanisms and future protocols.

## INTRODUCTION

Colorectal cancer (CRC) is a genetically heterogeneous group of oncological pathology involving uncontrolled cell growth with the ability to spread throughout the body causing tissue damage, intoxication, and severe pain [1]. It is commonly a lethal disease despite the enormous arsenal of treatments available on the market. In this commentary, we will discuss how the combinatorial therapy that we explored in our lab [2] could affect CRC cells resulting in strong suppression of cancer cell growth and what additional experiments might be necessary to support our recent results.

In recent years, several sets of experimental data have presented compelling evidence regarding the anti-oncogenic effects of cannabinoids on CRC [3–9]. There is a wide range of preclinical data indicating strong anticancer effects of cannabinoids on a variety of cancers including glioblastoma, meningioma, pituitary adenoma, endometrial sarcoma, prostate and colon carcinoma; however, in clinical settings, cannabinoids are currently used to alleviate chemotherapy-induced adverse effects (reviewed in [10]). The mechanisms responsible for the anticancer properties of cannabinoids, such as cannabidiol (CBD), include the initiation of apoptosis, activation of the endoplasmic reticulum (ER) stress response, suppression of survivin, and attenuation of RAS/MAPK and PI3K/AKT signalling pathways [3, 11, 12]. In CRCs, there is an overactivation of the RAS-MAPK pathway, with approximately 50% of cases exhibiting overexpression of KRAS and 15% showing overexpression of BRAF [13, 14]. Additionally, PI3K/AKT signalling is upregulated in nearly 40% of colon malignancies [15]. These pathways are of particular significance in our research because of their alterations in CRC and the observed impact of CBD on them.

Cancer cells face elevated levels of oncogene-induced genotoxic stress [16], oxidative damage [17], and metabolic stress [18], conditions typically absent in normal cells. Consequently, tumour cells heavily rely on stress-support pathways for their survival. Targeting these stress pathways, such as implementing fasting in cancer cells, could offer a more specific impact on tumours while preserving normal cells [19].

Studies demonstrated that *in vitro* serum starvation and *in vivo* short-term food deprivation led to decreased levels of growth factor stimulation [20–22]. In normal cells, the absence of growth signals results in reduced activity of proliferation-stimulating signalling and decreased metabolism [23, 24] Conversely, in cancer cells, starvation induces heightened cellular stress due to their metabolic reprogramming to sustain continuous proliferation [25] and triggers the activation of the DNA damage response [26]. Intermittent fasting could potentially enhance the sensitivity of certain tumours to chemotherapy drugs, including platinum drugs. This is attributed to the fact that, unlike normal cells, cancer cells struggle to adapt to fasting conditions, as they have accumulated mutations that promote cell growth specifically in conditions with great food (glucose) abundance. This phenomenon is known as differential stress sensitization [27]. Several rounds of starvation sensitized melanoma, glioma, neuroblastoma and breast cancer cells to chemotherapy [27]. Fasting is able to sensitize cancer cells to various chemotherapy agents, including mesothelioma and lung carcinoma cells to cisplatin [26] and pancreatic cancer xenograft to gemcitabine [28], hepatocellular cancer cells to sorafenib [29]. It should be noted that intermittent serum starvation (ISS) application may also prime cancer cells for better survival. Experiments demonstrated that serum starvation in prostate cancer cells triggers higher tolerance to oxidative stress and potentially higher survival when exposed to anti-tumorigenic agents [30]. As we previously noted, in CRC pathways that regulate cell metabolism like PI3K/AKT are often disrupted [15], which was our rationale for exploring the effects of fasting on CRC.

A study combining CBD with the platinum drug oxaliplatin demonstrated the ability of CBD to overcome oxaliplatin resistance [31]. Additionally, studies of drug resistance and cannabinoids suggest a potential chemosensitizing effect of cannabinoids in resistant CRCs [32–34]. Consequently, these observations influenced our decision to combine cisplatin, a widely used DNA-crosslinking platinum agent with CBD and ISS [2].

Overall, the metabolic changes observed in cancer cells present a potential target for anticancer therapies. As previously discussed, fasting induces metabolic shifts in cancer cells, resulting in differential stress sensitization. Furthermore, cannabinoids are recognized for their ability to modulate stress survival pathways in cancer cells. Therefore, the rational was to evaluate these treatments both independently and in combination to determine their cytotoxic effects on CRC cells and assess whether these treatments exhibit synergistic activity.

## SUMMARY OF THE ORIGINAL WORK AND CRITICAL ANALYSIS

In our original work [2], we tested CBD, ISS, and cisplatin alone as well as in different combinations such as – cisplatin and CBD; ISS and CBD; cisplatin and ISS; and cisplatin, CBD and ISS. The most prominent results in both drug interaction experiments and transcriptomic analysis were combinations between CBD and ISS, cisplatin and ISS, and finally, a combination of the three. Based on our research [2], the combination of CBD and ISS, as well as CBD, ISS and cisplatin, showed strong synergistic interactions in all tested CRC cell lines. However, a few other experimental designs could be explored.

First, we subjected cells to a 16-hour daily serum starvation regimen for five consecutive days. Our exploration was limited to this specific timeframe under serum-free conditions, as this timing was sufficient to inhibit cell growth by 50% in all tested cell lines. However, it would be interesting to investigate a broader range of timings for serum starvation. For example, testing the combination of CBD with ISS across various durations could help establish an optimal time window for synergistic interactions with CBD alone and in conjunction with cisplatin.

In addition, our experiments solely examined treatment conditions with a total absence of fetal bovine serum (FBS). It would be relevant to investigate a spectrum of FBS percentages incorporated into the treatments. For example, standard complete media typically includes 10% FBS. However, exploring variations such as 1%, 3%, 5%, and 7% FBS on CRC cell lines could help determine the specific range of FBS percentages contributing to the observed synergistic interactions. This approach would mimic so called fasting mimicking diet (FMD), which may be consider as more tolerable and forgiving, especially for elderly people with considerable frailty [35].

Moreover, the specific components absent in FBS responsible for the observed effects in our study remain unidentified. Therefore, we can not be certain it is the absence of carbohydrates that contributes to the synergistic effect. Thus, examining the absence of various components, such as different carbohydrates, albumins, hormones, and growth factors, among others present in FBS, would provide additional insights into the mechanistic connections underlying the synergistic effects of serum deprivation, CBD, and cisplatin. For example, it is established that lower protein content in FMD may be beneficial for sensitization of cancer cells to chemotherapy [36].

Finally, our drug interaction experiments were limited to three CRCs and one normal colon epithelial cell line, and we performed the transcriptomic analysis of treatments alone and in combinations only on one HCT-116 CRC cell line We chose HCT-116 due to the presence of the KRAS mutation, which represents a common mutation in CRCs. Expanding the scope of this experiment to include a greater number of cell lines from diverse cancers could provide insights into the susceptibility of other cancer types to our treatments. Transcriptomic profiling of a larger number of cell lines with different genomic backgrounds could also be done. Furthermore, conducting tests involving intermittent fasting, CBD, and cisplatin on animal models would offer additional information on the pathogenesis underlying the combinatorial treatments we employed and guide the implementation of these treatments with optimal efficiency in patients.

**Figure 1:**
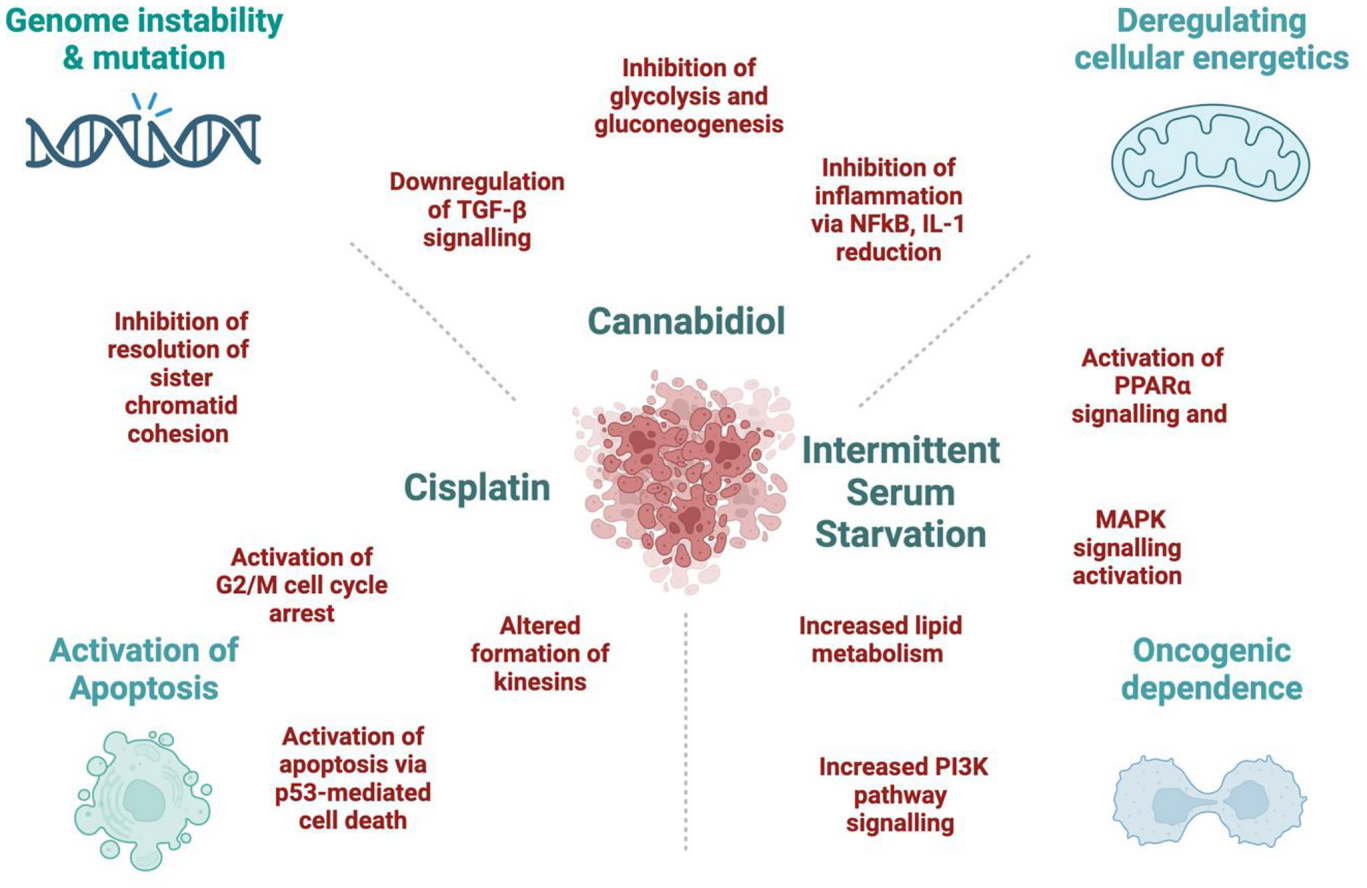
The potential mechanisms behind the synergistic effect between Cannabidiol, Cisplatin, and Intermittent Serum Starvation in the HCT-116 cell line. Figure created with https://www.biorender.com/.

In our work [2], cisplatin alone altered the genes associated with the transcription of p53-mediated apoptosis in the HCT-116 CRC cell line. It would be pertinent to reinforce these findings through apoptosis assays and the examination of protein expression related to genes responsible for cell death mechanisms. This approach would offer a more comprehensive understanding of the events occurring in cancer cells following cisplatin treatment. Moreover, when we added ISS to cisplatin treatment, we observed increased transcription of pro-survival pathways and lower enrichment of the p53-mediated cell death pathway. This could be a very important point for patients practicing fasting during chemotherapy to reduce chemotherapy-induced nausea and vomiting. Thus, having a better insight into these mechanisms could help in choosing a better approach for complex treatments of CRC patients and becoming more cautious about fasting during active chemotherapy. The potential mechanisms responsible for the synergistic effects are proposed in Figure 1.

Based on our data, CBD alone reduced the transcription of several genes linked to carbohydrate metabolism [2]. Nonetheless, our exploration of cancer cell metabolism remains limited. Investigating the mechanisms underlying the impact of CBD on CRC cell metabolism would constitute a crucial aspect of this research. Furthermore, we propose that these alterations were pivotal for the synergistic effect observed between CBD and ISS. Additionally, it was very interesting to observe that ISS alone changed the transcription of genes responsible for lipid metabolism and cell survival pathways in the HCT-116 CRC cell line. Thus, it would be pertinent to study PARPα and PARPγ signalling cascades since they heavily regulate lipid and carbohydrate metabolism and were affected under CBD and ISS treatments in our study.

Another aspect that could be introduced to these experiments involves testing various cannabinoids, including tetrahydrocannabinol or cannabinol, in addition to CBD, given that they too have demonstrated anticancer properties [3, 8, 12, 37]. Some studies reported that cannabinoid extracts can have higher potency over cannabinoids alone due to the entourage effects of different terpenes present in the cannabis plant [38, 39]. However, a potential drawback is that comparing the effects of cannabinoid extracts to purified cannabinoids in the context of the presented combinatorial treatments may introduce additional complexity from a pathogenetic standpoint. A viable solution could be to evaluate combinations of pure cannabinoids, terpenes, or other relevant molecules on the development of CRC.

## DISCUSSION AND FUTURE PERSPECTIVES

In 1926, Otto Warburg noted that cancer cells primarily produce ATP via the glycolytic pathway, even when oxygen is abundant [40]. The precise mechanisms driving the Warburg effect are still not fully understood. However, certain theories suggest that this adaptation allows cancer cells to divert resources toward biosynthesis while simultaneously reducing the production of reactive oxygen species (ROS) [41, 42]. The described metabolic changes in cancer cells offer potential avenues for anticancer therapy. Fasting induces alterations in the metabolism of cancer cells, leading to differential stress sensitization. Furthermore, cannabinoids have been recognized as regulators of stress survival pathways in cancer cells. Thus, combination of fasting, cannabinoids and chemotherapy may have better anti-cancer effect than chemotherapy alone.

A key hallmark of cancer is its rapid proliferation, frequently exposing cancer cells to conditions of hypoxia and limited nutrient availability. However, due to reduced levels of p53, apoptosis is not triggered as observed in normal cells [43]. The heightened proliferation of cancer cells demands an abundant supply of nutrients in the surrounding environment, as each cell cycle progression results in the generation of two daughter cells, necessitating a doubling of biomass [42]. Thus, in our experiment, introducing ISS alone reduced nutrient resources in cancer cells and slowed down their growth. Nevertheless, based on cell viability data, ISS alone was insufficient to induce cancer cell death [2].

Throughout cell division, the activation of the glycolytic pathway plays a crucial role in providing ATP and essential intermediates for various biosynthetic pathways. As a result, the Warburg effect contributes significantly to the overall enhancement of bioenergetics and biosynthesis [42]. On the contrary, inhibiting the glycolytic pathway in cancer cells might lead to stress overload due to continuous proliferative signals from oncogenes [44]. Tumors frequently encounter hypoxia-reperfusion events in their microenvironment, causing heightened production of free radicals, subsequently leading to mitochondrial damage and apoptosis [45]. As a response, tumours have adapted to alleviate oxidative damage by enhancing glycolysis and suppressing mitochondrial function [45]. We postulated that the substantial decrease in transcripts associated with the glycolytic pathway observed under CBD and ISS conditions in our experiments led to a pronounced suppression of the Warburg effect in CRC cells, ultimately resulting in extensive cell death.

Tumour cells display heightened glucose uptake, which enables cells to redirect accumulated pyruvate toward lipid biosynthesis, essential for membrane assembly [46]. The PI3K pathway, in conjunction with its downstream target AKT, stimulates glycolysis, leading to cell hypertrophy, increased glycolytic activity, and enhanced cell survival [47]. AKT can post-transcriptionally regulate various glycolytic steps, such as controlling the localization of glucose transporters such as GLUT1 in the cell membrane and activating hexokinase function, even in the absence of growth-stimulating signals. In light of our findings [2], we proposed a hypothesis where the synergistic interaction between ISS and CBD triggered an excessive activation of PI3K/AKT signalling. Consequently, cancer cells were compelled to rely on glycolysis. Conversely, CBD exhibited a robust inhibition of carbohydrate metabolism. As a result, cancer cells encountered difficulties in generating adequate ATP through pro-survival pathways, ultimately resulting in the upregulation of cell death transcripts. We strongly believe that further comprehensive investigation of this combined treatment is warranted, as a simple integration of intermittent fasting with CBD has the potential to significantly impact patient outcomes with reduced adverse effects.

Another part of our experiments involved the addition of cisplatin to combinatorial treatments. Cisplatin exhibits a potent pro-apoptotic effect [48, 49], aligning with our findings in the HCT-116 CRC cell line. The cytotoxicity of cisplatin is attributed to its capability to create DNA crosslinks, leading to DNA damage, and triggering apoptosis [50]. Unfortunately, the frequent occurrence of resistance to cisplatin and, consequently, cancer progression is a notable challenge [51, 52]. Based on the mRNA expression data acquired from the HCT-116 CRC cell line, cisplatin downregulated multiple metabolic pathways crucial for the growth and proliferation of cancer cells. Simultaneously, cisplatin induced cytotoxic effects by upregulating the expression of several genes involved in both the intrinsic and extrinsic pathways of apoptosis. Still, there was also an increase in transcripts responsible for cell survival and cancer cell resistance. To validate the concurrent occurrence of cisplatin’s cytotoxic effects and the emergence of cancer cell survival mechanisms, we recommend conducting single-cell transcriptomic and genomic analyses. This approach would unveil the evolution of subclones resistant to the action of cisplatin.

Based on our results, we proposed that ISS and CBD, by suppressing metabolic pathways in the HCT-116 CRC cell line, contributed to the cytotoxic effects of cisplatin, leading to the upregulation of pro-apoptotic genes and G2/M arrest, which resulted in a strong synergistic effect. It would be intriguing to investigate whether this combination could induce mitotic catastrophe and cell death across various CRC cell lines.

In summary, our study was limited to cell viability and mRNA expression to unravel treatment synergies within CRC cell lines, providing initial insights into the underlying molecular mechanisms of ISS, CBD and cisplatin interactions (Figure 1). Nevertheless, further investigations are necessary to substantiate our claims. Our effort establishes the foundation for subsequent explorations in this field. We recognize that the primary limitation of our study lies in the reliance on analyses of cell viability and mRNA expression. To validate our hypotheses, further confirmatory studies are essential, including protein-level analyses and pathway enrichment assessments. Additionally, expanding the range of cell lines tested and incorporating *in vivo* studies would provide crucial insights into the pathogenesis and the underlying rationale for the proposed combinatorial treatments. These aspects are being further investigated through ongoing experimental work in our lab.
